# Human microbiota: ‘The philosophers have only interpreted the world in various ways. The point, however, is to change it’.

**DOI:** 10.1111/1751-7915.12259

**Published:** 2015-01-28

**Authors:** Harald Brüssow

**Affiliations:** Department Human Microbiology Group, Nestlé Research Center, Nutrition and Health DepartmentCH-1000, Lausanne, 26, Switzerland

Even though we still do not know the ultimate number of bacterial species in the world, our understanding of bacteria populating the human body has increased enormously with the Human Microbiome Project. Act One (the Exposition in classical theatre) of this international effort – in describing what is colonizing our bodies – is now coming if not to an end, then to a saturation phase. Scientists already investigate statistical associations between microbiota composition and health and disease conditions (Act Two, the Complication phase). Many of these investigations took the form of case–control studies, such as gut microbiota in patients with liver cirrhosis and matched healthy controls, to quote a recent example (Qin *et al*., 2014). In the next years, we will see many more of such case–control studies. They will provide us data where a human microbiota dysbiosis is associated with a disease or an undesired physiological condition. Such data, if confirmed in independent studies with different human populations, will identify disease states that are strongly associated with microbiota disequilibria. However, the old epidemiological dictum that association is not causation also applies to these studies. It has rightly been mentioned that many such studies suffer from reverse causation artefacts (Hanage, 2014). In the future, we will therefore see more prospective human microbiota studies defining whether the microbiota change precedes the development of the deteriorating health condition or is a consequence of this change, bringing us to Act Three, the Reversal phase. This temporal argument is a strong discriminator for health changes induced or followed by microbiota upsets. Although still not very numerous, this research is already on the stage: dynamic studies on age-related microbiota changes in elderly people transferred to residence homes (Claesson *et al*., 2012) and infants suffering from malnutrition are examples (Subramanian *et al*., 2014). However, prospective studies are still no guarantee for causal relationships. This argument can only be provided by intervention studies. In fact, a sentence that was formulated by Karl Marx as a mid-19th century critique of classical philosophy also applies to the microbiota field: ‘The philosophers have only interpreted the world in various ways. The point, however, is to change it’. We have already seen very successful examples for such interventions: Act Four, the Retardation phase, is already being played. The most striking example is the application of processed stool samples from healthy donors to patients suffering from *Clostridium difficile* infections (van Nood *et al*., 2013). This form of bacteriotherapy led to rapid and dramatic ameliorations of the clinical condition in these patients. Antibiotics in treating malnutrition in children is another, although more controversial case (Trehan *et al*., 2013). Bacteriotherapy with processed faeces and antibiotics are, however, rather broad spectrum interventions unlikely to please regulatory authorities.

We are therefore waiting for Act Five, which classically brings the Lysis or Resolution of the drama. More specific interventions correcting microbiota disequilibria are thus needed. Phage therapy targeting a single bacterial species for lysis might be an option to remove or decrease undesired bacteria, but controlled clinical trials proving its value are still largely lacking. Health-promoting probiotic bacteria have shown some success in a few well-defined areas of gastroenterology, vaginal health and allergy. However, introducing probiotic bacteria into an already highly colonized niche, such as the intestine, meets colonization resistance from the resident gut microbiota, even if not all probiotic effects depend on colonization. An alternative approach is feeding prebiotics – food that passes the human intestine unchanged and becomes nutrients for selected parts of the resident gut microbiota. This approach already increased bifidobacteria in human faeces after feeding oral bifidogenic oligosaccharides. Defining other prebiotic candidates (e.g. resistant starch) has started, but *in vitro* identification is complicated by the fact that dietary fibre degradation in the gut is frequently the work of cooperating bacterial species necessitating gut models with complex microbiota as test systems. However, nature might provide us a potential lead. Lactating mothers produce large amounts of oligosaccharides into the breast milk, which are not digested by the infant's gut. As this biosynthetic investment of mothers would not make much sense evolutionary, researchers have searched biological functions for human milk oligosaccharides (HMO) (Smilowitz *et al*., 2014). One interpretation is that HMO act as decoys for gut pathogens, preventing them from docking on receptors of the gut epithelia. This hypothesis could explain the large chemical variability of HMO. An alternative interpretation is that HMO instruct the infant's gut which bacteria should colonize by favouring those able to digest HMO (Zivkovic *et al*., 2011). In this scenario, the first step of what happens naturally would be seeding of the newborn with a desired maternal microbiota from the vagina (during delivery), the mouth (kissing) and skin (breast feeding). The second step is by providing naturally designed prebiotics that increase the proportion of a physiologically desired microbiota to assist the many microbiota-induced maturation processes in the developing infant gut. The strong *in vitro* bifidogenic effect of breast milk and the prominent bifidobacterial colonization of the gut in breast-fed babies would fit to this interpretation. Chemical synthesis of oligosaccharides and their biotechnological production have developed substantially over the last years and one might therefore expect to soon see feeding trials with such synthetic prebiotics in infants (Bode, 2012). As the rate of hospital deliveries, Caesarean sections and formula feeding is high in most developed countries, and likely to increase in threshold countries, synbiotics (combinations of a desired set of health-promoting gut microbes together with their specific nutritional growth promoters as prebiotics) might appear in the crystal ball as routine in hospital deliveries and in subsequent infant formula feeding where mothers do not opt for the natural solution (home delivery and breast feeding). Such a symbiotic could fend off colonization of the newborn with hospital germs and promote a desired gut microbiota establishment with its suspected positive impact on the maturation of the developing infant gut and immune system.

## Conflict of interest

None declared.
